# Epigenetics Glossary

**Published:** 2013

Adipogenesis: Process of cell differentiation by which fat cells (i.e., adipocytes) are generated from precursor cells.
AMP/ATP ratio: Ratio of adenosine monophosphate (AMP) to adenosine triphosphate (ATP) in the cell; ATP supplies the energy needed for many biochemical reactions; therefore, the AMP/ATP ratio reflects the cell’s energy level.
Apoptosis: Series of biochemical reactions occurring in a cell whereby cells that are damaged or no longer needed undergo a process of self-destruction; also known as programmed cell death or cell suicide.
Astrocytes: Characteristic star-shaped *glial cells* in the brain and spinal cord that support the endothelial cells which form the blood–brain barrier, provide nutrients to the nervous tissue, and play a principal role in the repair and scarring process of the brain and spinal cord following traumatic injuries.
Caloric restriction: A dietary regimen characterized by a low calorie intake while maintaining adequate nutrition (i.e., sufficient levels of proteins, vitamins, and minerals).
Cerebellum: Region of the brain that controls motor function and plays a role in sensory perception.
Centromere: The central part of a chromosome where the two “arms” of the chromosome are attached to each other.
Competitive inhibitor: Any molecule that can bind to a receptor or an enzyme and prevent binding of the molecule that normally interacts with the receptor or enzyme, thereby inhibiting normal receptor or enzyme function.
Electron transport chain: An electron transport system located in the *mitochondria*, in which electrons released by *NADH* are passed on to a series of other molecules that first accept the electrons and then pass them on to the next molecule in the chain. The electrons ultimately are transferred to oxygen to generate water. These successive reactions provide enough energy to drive the synthesis of ATP molecules.
Endoparasitic sequences: DNA sequences that are repeated multiple times in the genome; a type of *transposable element*.
Endoplasmic reticulum: A system of folded membranes in the cell that loop back and forth, spreading throughout the cytoplasm and providing a large surface area for cell reactions.
Epigenome: Entirety of all epigenetic changes in a cell, tissue, or organism.
Folate: Vitamin B9; is needed by the body to synthesize, repair, and methylate DNA; alcohol consumption can lead to folate deficiency.
Folate cycle: A series of biochemical reactions in which one-carbon units (e.g., methyl groups) are transferred from folate or, more specifically, its derivative tetrahydrofolate, to other molecules.
Genotype: The complete genetic makeup of an individual organism that is determined by the specific variants (i.e., alleles) of each gene carried by the individual. Differences in alleles among individuals account for the differences in *phenotype* observed among those individuals.
Glial cells: Cells that provide support and protection for neurons.
Glutathione (GSH): An antioxidant molecule found naturally in the body, composed of three amino acids (i.e., glutamate, cysteine, and glycine).
Heterozygous: Carrying two different variants (i.e., alleles) of a given gene.
Hippocampus: Brain region shaped like a curved ridge found within the cerebral hemisphere that functions in consolidation of new memories; also thought to play a role in alcohol withdrawal seizures.
Homeostasis: The maintenance of a stable internal state or condition (e.g., body temperature or blood pressure) in a living organism.
Homozygous: Carrying two copies of the same variant (i.e., allele) of a given gene.
Hypoglycemia: Lower-than-normal blood sugar levels.
Hypomethylation: Lower-than-normal levels of epigenetic methylation of the DNA.
Imprinting: A genetic phenomenon by which certain genes are marked by biochemical modifications after conception so that only the gene copy inherited from one of the parents is expressed whereas the imprinted gene copy is silenced; for example, for certain genes only the copy inherited from the father will be expressed.
Microglia: Type of *glial cell* that acts as the first and main form of active immune defense in the central nervous system.
Microsatellite: Highly variable DNA regions found every few thousand nucleotides in the DNA that can be used to determine from which parent or ancestor a specific DNA sequence has been inherited. Micro-satellites typically consist of short sequences of 1 to 6 nucleotides that can be repeated 10 to 100 times. Each person or animal has a specific pattern of micro-satellites that can be used to determine inheritance patterns.
Mitochondria: Structures within cells that generate most of the cells’ energy through the production of adenosine triphosphate (ATP), a molecule that provides the energy needed for many key metabolic reactions.
Messenger RNA (mRNA): Key intermediary molecule generated when a gene is expressed (i.e., when the infor mation encoded in the gene is converted into a protein product by the cell); mRNA levels for a gene are used as an indicator of how “active” the gene is (i.e., how much of the protein is produced).
Myelin: A white fatty material composed chiefly of alternating layers of lipids and lipoproteins that encloses the long extensions (i.e., axons) of myelinated nerve fibers.
Nicotinamide adenine dinucleotide (NAD): NAD is a molecule that binds with hydrogen atoms and becomes reduced NAD (NADH), during alcohol metabolism and other chemical reactions in the cell. NAD and NADH move hydrogen atoms back and forth with other molecules in the cell, thus helping to maintain balance between oxidation and reduction in the cell.
NADH/NAD^+^ ratio: The ratio of reduced *NAD* to oxidized *NAD*; changes in this ratio can promote or interfere with certain biochemical reactions that require either NAD^+^ or NADH as cofactors.
Neoplastic transformation: Process by which normal cells are transformed into malignant tumor cells.
Non-coding RNA (ncRNA): RNA molecules that are not used as a template to produce proteins.
Oncogene: A gene that has the potential to cause cancer, especially if it becomes mutated or expressed at high levels.
One-carbon metabolism: Network of biochemical reactions in which a chemical unit containing one carbon atom (e.g., a methyl group) is transferred through several steps from a donor to another compound, such as DNA.
Orthologue: Genes in different species that evolved from a common ancestral gene by speciation. Normally, orthologue genes retain the same function throughout the course of evolution.
Oxidative stress: An imbalance between oxidants (e.g., *reactive oxygen species [ROS]*) and antioxidants (e.g., *glutathione [GSH]*) that can lead to excessive oxidation and cell damage.
Phenotype: The observable structural or functional characteristics of an individual organism that result from the interaction of its genetic makeup (i.e., *genotype*) with environmental factors.
Polymorphism: The presence of two or more variants (i.e., alleles) of a gene or other DNA sequence in a population.
Promoter: A DNA segment located at the start of a gene’s coding sequence that provides a binding site for the enzymes that initiate the first step in the process of gene expression (i.e., *transcription*).
Quantitative trait locus (QTL): A DNA region that is associated with a quantitative trait—a *phenotype* that varies in the degree to which it is present (e.g., sensitivity to alcohol or height) and which typically is determined by more than one gene—and which may contain one of the genes contributing to that trait.
Reactive oxygen species (ROS): Highly reactive oxygen– containing free radicals that are generated during oxidative metabolism. ROS can react with and damage lipids, proteins, and DNA in cells, causing *oxidative stress*. Common ROS include hydrogen peroxide, superoxide radicals, and hydroxyl radicals.
Recombinant inbred strains: Sets of animal strains that all are derived from the same two parental inbred strains and which each carry a specific combination of the parental genes; within each RI strain all animals are genetically identical.
Redox/Redox state: Shorthand for reduction/oxidation reactions. The term redox state is often used to describe the balance of *NAD* and NADH in a biological system such as a cell or organ. An abnormal redox state can develop in a variety of deleterious situations.
Retrotransposon: DNA segment that can duplicate itself and thus multiply in the genome; during this process, the original DNA sequence first copies itself into RNA and then back into DNA, which is then incorporated back into the genome; retrotransposons make up a substantial portion of the genome.
RNA splicing: The removal of noncoding sequences (i.e., introns) from the sequence of an *mRNA* following *transcription* to form an uninterrupted coding sequence.
S-adenosylmethionine (SAM): Compound that serves as the principal donor of methyl groups for methylation reactions.
Somatic mutation: Alterations of the DNA that occur after conception in any of the cells of the body except the germ cells and which therefore cannot be passed on to offspring.
Substrate: A molecule that is acted upon by an enzyme.
Teratogenesis: The development of malformations or defects in a developing embryo or fetus.
Transcription: Biochemical process in which an intermediary molecule called *messenger RNA* is generated based on the genetic information of the DNA.
Transcription factor: Protein regulating the *transcription* of a gene; consists of at least two functional domains: a DNA-binding domain and an activating domain.
Transposable elements: A DNA segment that can change its position within the genome; *retrotransposons* are a type of transposable element.
Tricarboxylic acid (TCA) cycle: A series of biochemical reactions that serve to generate energy from the metabolism of acetyl-CoA, which in turn is derived from the metabolism of sugars, fats, and proteins; also called citric acid cycle.

